# Performance of Diagnostic Tests for Histoplasmosis Across Clinical Syndromes and Immune Statuses

**DOI:** 10.1093/ofid/ofaf688

**Published:** 2025-11-12

**Authors:** Nicolas Barros, Mitch McClean, Elisa Sarmiento, Shanna Noveroske, John Witt, L Joseph Wheat

**Affiliations:** Department of Medicine, Indiana University School of Medicine, Indianapolis, Indiana, USA; Miravista Diagnostics, Indianapolis, Indiana, USA; Department of Medicine, Indiana University School of Medicine, Indianapolis, Indiana, USA; Department of Medicine, Indiana University School of Medicine, Indianapolis, Indiana, USA; Miravista Diagnostics, Indianapolis, Indiana, USA; Miravista Diagnostics, Indianapolis, Indiana, USA; Miravista Diagnostics, Indianapolis, Indiana, USA

**Keywords:** antibody, antigen, diagnosis, histoplasma, histoplasmosis

## Abstract

**Background:**

Histoplasmosis, caused by *Histoplasma capsulatum*, presents with a broad clinical spectrum from asymptomatic infection to disseminated disease (DH). Diagnostic modalities, culture, histopathology, antigen detection, and serologic testing, vary in performance. We evaluated their sensitivity and specificity across clinical presentations and immune statuses.

**Methods:**

We conducted a retrospective diagnostic study of adults evaluated at Indiana University Health Medical Center who had serum and/or urine submitted for Histoplasma antigen testing to Miravista Diagnostics. Clinical classification (proven, probable) was based on laboratory, imaging, and clinical data according to EORTC/MSG criteria. Controls were patients tested for histoplasmosis but found to have an alternative diagnosis. Diagnostic methods included antigen enzyme immunoassay (EIA), antibody EIA (IgG/IgM), immunodiffusion (ID), complement fixation (CF), and fungal cultures.

**Results:**

Among 205 patients, 125 were classified as histoplasmosis (54 proven, 71 probable) and 80 as controls. Antigen EIA in serum and/or urine had sensitivity 121/124 (97.6%) and specificity 75/80 (93.7%). IgG/IgM antibody EIA detected 89/111 (80.2%) cases with specificity 73/79 (92.4%) at a 20 EU/mL cutoff. Combining antigen and antibody testing increased sensitivity to 109/110 (99.1%). Among 75 patients with both serum and urine antigen tested, combined testing added 3/75 (4%) cases beyond urine alone. Antibody sensitivity declined in immunocompromised patients (75.0% vs 93.3% in immunocompetent, *P* = .0134), while antigen sensitivity remained high across groups.

**Conclusions:**

Histoplasma antigen testing is the most sensitive diagnostic method. Antibody testing complements antigen in selected populations, particularly immunocompetent patients with pulmonary disease. Culture remains a complementary but less sensitive tool.

## Teaser

Antigen (urine or serum) is highly sensitive; combining assays yields modest incremental benefit and may be useful in select contexts. Antibody EIA is most helpful in immunocompetent patients with subacute pulmonary disease. Understanding test performance by immune status and disease stage can guide clinicians in selecting diagnostic strategies and interpreting results.

## BACKGROUND

Histoplasmosis is among the most common dimorphic fungal infections in the Americas. It is caused by the infection with *Histoplasma capsulatum*, a thermally dimorphic fungus that exists as mold in the environment and converts to yeast at body temperature [1]. While most infections are self-limiting, the disease spectrum ranges from acute pulmonary histoplasmosis (APH) to subacute (SPH) and chronic pulmonary histoplasmosis (CPH), as well as disseminated histoplasmosis (DH) [2].

Diagnosis of histoplasmosis can be challenging and relies on multiple methods, each with limitations [3]. The gold standard, fungal culture or histopathologic identification, lacks sensitivity, and in the case of cultures, it also requires prolonged incubation. Serologic testing, including immunodiffusion (ID) and complement fixation (CF) assays, is widely used, but is limited by low sensitivity, especially in those who are immunocompromised. The detection of antibodies by enzyme immunoassay (EIA) offers increased sensitivity over current antibody tests while also allowing separate detection of IgG and IgM antibodies, though it is not widely available [4].

Antigen detection significantly improved diagnosis by offering a rapid, noninvasive, and highly sensitive method, particularly for DH. Refinements in EIAs have improved specificity and allowed for antigen quantification [5]. When urine and serum antigen tests are combined, the sensitivity exceeds 80% in APH [4].

This study aimed to evaluate the sensitivity and specificity of diagnostic tests (culture, histopathology, antigen detection, and serologic testing) across various clinical syndromes and levels of immunosuppression in a tertiary referral center.

## METHODS

### Patient Samples

Adults (≥18 years) evaluated at Indiana University Health Medical Center, Indianapolis who had serum and/or urine submitted to Miravista Diagnostics (MVD) for antigen testing and had residual sample stored for further evaluations.

### Data Collection

Medical record review was performed to obtain patient demographics including age, gender, comorbidities, presence of immunosuppression or immunocompromising conditions, type (acute pulmonary, pulmonary, or extrapulmonary) and severity of disease presentation, results of diagnostic tests, and treatment. Clinical data, radiographic imaging, laboratory findings, and diagnosis were reviewed to assess if patients met the diagnostic classifications of currently active histoplasmosis based on the EORTC/MSG definitions criteria [6]. Data were collected under a uniform case report. Patients diagnosed with other invasive fungal infections were excluded from the analysis.

The protocol was reviewed and approved by the Indiana University institutional review board (IRB–15195) prior to implementation.

### Case Definition

Diagnostic criteria for histoplasmosis included a positive result of culture, antigen, histopathology, or cytopathology in combination with compatible clinical and radiographic findings. Anti-*Histoplasma* antibodies by ID, CF, or EIA were not considered for inclusion.

### Case Classification

#### Proven

Presence of positive culture, cytology, or histopathology demonstrating yeast-like structures characteristic of *Histoplasma capsulatum.*

#### Probable

Positive antigen in combination with compatible clinical and radiographic findings.

### Controls

Patients in whom histoplasmosis was considered in the differential diagnosis but who were ultimately confirmed to have an alternative diagnosis accounting for their presenting syndrome.

### Clinical Classification

#### Disseminated Histoplasmosis

Presence of clinical, imaging, or laboratory evidence of extrapulmonary involvement.

#### Pulmonary

Presence of respiratory symptoms in association with pulmonary imaging demonstrating pulmonary infiltrates and/or mediastinal lymphadenopathy without evidence of DH. APH was defined by symptom duration <1 month; SPH was defined by symptom duration ≥1 month but <3 months, and CPH was defined by symptom duration ≥3 months with presence of cavitary pulmonary infiltrates.

### Disease Severity

#### Mild Disease

Defined as disease requiring outpatient care only.

#### Moderate Disease

Defined as requiring hospitalization but no admission to intensive care unit.

#### Severe Disease/Death

Defined as requiring management in intensive care unit or resulting in death.

### Statistical Analysis

Statistical analysis was performed using SAS version 9.4 (SAS Institute Inc., North Carolina, USA). χ^2^ analysis was used to compare unpaired samples. The McNemar test was used to compare diagnostics assays using paired samples. Sidak correction was used to adjust for multiple comparisons. The *t*-test or ANOVA were used to compare continuous variables. *P*-values < .05 are considered significant. Figures were performed in Prism GraphPad version 10.0.2 (GraphPad Software, San Diego, California USA).

## RESULTS

### Study Population

A total of 205 individuals were included; 125 individuals were classified as cases (54 proven and 71 probable), and 80 were classified as controls (Table 1). There were 75 cases with pulmonary involvement, and of these 49 cases were acute, 16 cases were subacute, and 5 cases were chronic (5 had incomplete data to assess chronicity). Thirty-three cases had disseminated disease, 11 had CNS involvement, and 6 had extrapulmonary manifestations that could not be clearly categorized. When classified by severity of disease, 24 had mild disease managed as outpatient, 74 had moderate disease requiring inpatient admission, 17 had severe disease requiring critical care management, and 10 died.

**Table 1.** Population Characteristics, *N* = 205

	Cases	Controls	*P-*value
	*N* = 125 (60.98)	*N* = 80 (39.02)	
Demographics, *N* (%)			
Gender			.54004
Female	57 (45.6%)	33 (41.3%)	
Male	68 (54.4%)	47 (58.7%)	
Race			.0061
White	105 (85.4%)	69 (87.3%)	
Black or African American	14 (11.4%)	5 (6.3%)	
Hispanic or Latino	0 (0%)	5 (6.3%)	
Asian	4 (3.2%)	0 (0%)	
Comorbidities			
Autoimmune disorder	49 (39.2%)	11 (13.8%)	.0001
Diabetes	14 (11.2%)	18 (22.5%)	.0297
Chronic liver disease	9 (7.2%)	13 (16.3%)	.0411
CKD	21 (16.8%)	11 (13.8%)	.5573
Lung disease	13 (10.4%)	7 (8.8%)	.6977
Immunocompromised	91 (72.8%)	36 (45.0%)	<.0001
HIV/AIDS	11 (8.8%)	2 (2.5%)	.071
SOT	23 (18.4%)	9 (11.3)	.1689
Heme/Onc	6 (4.8%)	10 (12.5%)	.045
Oncology	4 (3.2%)	8 (10.0%)	.0646
Medications, *N* (%)			
Immunosuppressive medications	76 (60.8%)	25 (31.3%)	<.0001
Steroids	20 (16.0%)	7 (8.8%)	.1343
TNFa inhibitor	32 (25.6%)	5 (6.3%)	.0004

Of the 125 confirmed cases, 91 had immunocompromising conditions including 23 with solid organ transplant, 11 with advanced HIV, 6 with hematologic malignancy, 4 with nonhematologic malignancy, and 47 with other types of immunosuppression. 76 of the cases were taking immunosuppressive medications at time of diagnosis, 32 were taking TNF-alpha inhibitors, 20 were taking corticosteroids, 15 were taking calcineurin inhibitors, and the rest were taking other immunosuppressing medications (cytotoxic agents, chemotherapy or other biological agents).

Of the 80 controls, 30 had immunocompromising conditions including 9 with solid organ transplants, 2 with advanced HIV, 10 with hematologic malignancy, 8 with nonhematologic malignancy, and 1 with another type of immunosuppression. Of the 25 controls taking immunosuppressive medications at time of testing, 5 were taking TNF-alpha inhibitors, 7 were taking corticosteroids, 6 were taking calcineurin inhibitors, and the rest were taking other immunosuppressing medications (cytotoxic agents, chemotherapy or other biological agents).

### Sensitivity and Specificity

Characteristics for each diagnostic test for all cases of histoplasmosis and controls are shown in Table 2.

**Table 2.** Overall Performance of Different Tests for the Diagnosis of Histoplasmosis

	Sensitivity	Specificity	LR Positive	LR Negative
MVD Ag EIA				
Urine	93/98 (94.9% CI 88.6–97.8)	41/44 (93.2% CI 85.7–100.0)	13.92	0.05
Serum	96/101 (95.1% CI 90.8–99.3)	73/76 (96.1% CI 91.7–100.0)	23.84	0.06
Serum and/or urine	121/124 (97.6% CI 94.9–100)	75/80 (93.7% CI 88.3–100.0)	15.29	0.03
BAL	19/23 (82.6% CI 67.1–98.1)	11/11 (100.0% CI 74.1–100.0)	…	0.17
MVD Ab EIA (>10 EU/mL)				
IgG	80/111 (72.1% CI 63.7–87.3)	69/79 (87.3% CI 80.0–94.7)	5.64	0.33
IgM	74/111 (66.7% CI 57.9–75.4)	64/79 (81.0% CI 72.4–89.7)	3.48	0.42
All	89/111 (80.2% CI 72.8–87.6)	57/79 (72.2% CI 62.3–82.0)	2.86	0.28
MVD Ab EIA (>20 EU/mL)				
IgG	76/111 (68.5% CI 59.8–77.1)	75/79 (94.9% CI 90.1–99.8)	13.40	0.33
IgM	52/111 (46.9% CI 37.6–56.1)	76/79 (96.2% CI 92.0–100.0)	12.22	0.56
All	89/111 (80.2% CI 72.8–87.6)	73/79 (92.4% CI 86.6–98.3)	10.46	0.22
MVD Ag and/or Ab EIA (>10 EU/mL)	109/110 (99.1% CI 97.3–100)	54/80 (67.5% CI57.2–77.8)	3.02	0.02
MVD Ag and/or Ab EIA (>20 EU/mL)	109/110 (99.1% CI 97.3–100)	71/80 (88.8% CI 81.8–95.7)	8.61	0.02
ID (H/M Ab)				
H band	21/66 (31.8% CI 20.6–43.1)	34/34 (100.0% CI 89.9–100.0)	…	0.67
M band	37/66 (56.1% CI 44.1–68.0)	32/34 (94.1% CI 86.1–100.0)	9.53	0.47
All	39/66 (59.1% CI 47.2–71.0)	32/34 (94.1% CI 86.1–100.0)	7.08	0.44
CF (Yeast/Mycelial)				
Mycelial	18/52 (34.6% CI 21.7–47.6)	21/21 (100.0% CI 84.5–100.0)	…	0.66
Yeast	19/51 (37.3% CI 24.0–50.5)	20/21 (95.2% CI 86.1–100.0)	7.67	0.66
All	23/52 (44.2% CI 30.7–57.7)	20/21 (95.2% CI 86.1–100.0)	9.44	0.57
Culture				
Blood	2/89 (2.3% CI 0.0–5.3)	52/52 (100.0% CI 93.1–100.0)	…	0.98
Fugal blood	16/31 (51.6% CI 34.8–68.0)	7/7 (100.0% CI 64.6–100.0)	…	0.48
Respiratory (sputum)	2/28 (7.1% CI 1.3–22.7)	25/25 (100.0% CI 86.7–100.0)	…	0.93
BAL	22/40 (55.0% CI 39.8–69.3)	14/14 (100.0% CI 78.5–100.0)	…	0.45
Pathology				
Histopathology	22/36 (61.1% CI45.2–77.0)	19/19 (100.0% CI 83.2–100.0)	…	0.41
Cytopathology	13/38 (34.2% CI 19.1–49.3)	18/18 (100.0% 82.4–100.0)	…	0.66

Abbreviation: CI, confidence interval.

Antigen detection by EIA in serum and/or urine identified 121/124 cases (97.6% CI 94.9–100) and was more sensitive than antibody detection by EIA at a cutoff of 10 EU/mL or 20 EU/mL (89/111, 80.2% CI 72.8–87.6 *P* < .0001), ID (39/66, 59.1% CI 47.2–71.0 *P* < .0001) and CF (23/52, 44.2% CI 30.7–57.7 *P* < .0001). The specificity of antigen detection by EIA in serum and/or urine was 75/80 (93.7% CI 88.3–100) and was more specific than antibody detection by EIA at a cutoff of 10 EU/mL (58/80, 72.2% CI 62.3–82.0 *P* = .0004) but was similar to antibody detection by EIA at a cutoff of 20 EU (74/80, 92.4% CI 86.6–98.3 *P* = .7389), ID (77/82, 94.1% CI 86.2–100 *P* = .5637) and CF (79/83, 95.2% CI 86.1–100 *P* = .0833).

Only 75 cases had both urine and serum performed at the same time. Among them, 68 were positive for both tests, 3 were serum only, 2 urine only, and 2 negative on both. Combined serum and urine antigen testing therefore added 3/75 (4%) cases compared with urine alone.

Antibody detection by EIA (both IgM and/or IgG) had higher sensitivity (89/111, 80.2% CI 72.8–87.6) when using cutoff of EU/mL >10 or EU/mL >20 when compared to ID (39/66, 59.1% CI 47.2–71.0 *P*  *=* .0005 and *P* = .0013, respectively) and CF (23/52, 44.2% CI 30.7–57.7 *P* = .0006 and *P* = .0016, respectively). The specificity of antibody detection by EIA at a cutoff of 10 EU/mL was 57/79 (72.2% CI 62.3–82.0) and 73/79 (92.4% CI 86.6–98.3) at a cutoff of 20 EU/mL. The specificity at 10 EU was lower when compared to ID (32/34, 94.1% CI 86.2–100 *P*  *=* .016) and CF (20/21, 95.2% CI 86.1–100 *P*  *=* .0196). However, the specificity at a cutoff of 20 EU/mL was similar to ID (*P* = .3173) and CF (*P* = .5637).

Testing for both antigen and antibody by EIA had a sensitivity of 109/110 (99.1% CI 97.3–100) at either cutoff. The sensitivity was similar to antigen detection in serum and/or urine (cutoff of 10 EU/mL and 20 EU/mL *P* = .1573 and *P* = .1573, respectively) but higher than ID (cutoff of 10 EU/mL and 20 EU/mL *P* < .0001, *P* < .0001, respectively) or CF (cutoff of 10 EU/mL and 20 EU/mL *P* < .0001, *P* < .0001, respectively). The specificity of testing both antigen and antibody by EIA was 54/79 (68.4% CI 57.2–77.8) using an antibody EIA cutoff of 10 EU/mL and 70/79 (88.6% CI 81.8–95.7) using a cutoff of 20 EU/mL. The specificity, using the cutoff of 10 EU/mL was lower than antigen in urine and/or serum (*P* < .0001), ID (*P* = .0009), and CF (*P* = .0027). However, when the cutoff was increased to 20 EU/mL, the specificity was similar to that of antigen in urine and/or serum (*P* = .1025), ID (*P* = .1573), or CF (*P* = .0833).

Blood cultures had the lowest sensitivity with only 2/89 (2.2% CI 0–5.3) of the tests being positive while using fungal isolators for blood cultures had a sensitivity of 16/31 (51.6% CI 34.8–68.0). The median time to positivity for fungal blood cultures was 22.5 +/− 8.1 days. Sputum cultures had a sensitivity of 2/28 (7.1% CI 1.3–22.7) but fungal cultures from bronchoalveolar lavage had a sensitivity of 22/40 (55% CI 39.6–70.4)

### Immunocompetent and Immunocompromised Individuals

When stratified by immunocompromised status (Table 3), in the immunocompromised population, combined antigen testing of both serum and urine had the highest sensitivity 91/91 (100.0% CI 96.0–100.0). Serologic testing for antibodies was less sensitive in immunocompromised patients compared to immunocompetent ones. IgM/IgG antibodies by EIA at a cutoff of 10EU/mL were positive in 26/27 (93.3% CI 81.7–99.8) of immunocompetent individuals versus 63/84 (75.0% CI 64.8–83.0) of immunocompromised patients (*P* = .0134). Increasing the threshold to 20 EU/mL had a minimal impact on the sensitivity of 24/27 (88.9% CI 71.9–96.2) in immunocompetent individuals versus 57/84 (67.9% CI 57.3–76.9) (*P* = .0449). The sensitivity of antibody detection by ID was 15/21 (71.4% CI 50.0–56.2) in immunocompetent individuals versus 24/45 (53.3% CI 39.1–67.1) in immunocompromised individuals (*P* = .1637). The sensitivity of antibody detection by CF was 5/14 (40% CI 50–86.2) among immunocompetent individuals and 18/38 (47.4% CI 32.5–62.7) among immunocompromised individuals (*P* = .5388).

**Table 3.** Differences in Sensitivity Between Immunocompromsied and Immunocompetent Individuals

	Immunocompromised				Immunocompetent	*P-*value^a^
	SOT	HIV AIDS	TNF-α inhibitor	All		
MVD Ag EIA						
Urine	21/21 (100.0% CI 84.5–100.0)	9/10 (90.0% CI 59.6–99.5)	31/31 (100.0% CI 89.0–100.0)	77/79 (97.5% CI 91.2–99.6)	16/19 (84.2% CI 62.4–94.5)	.0485
Serum	18/18 (100.0% CI 82.4–100.0)	8/8 (100.0% CI 67.6–100.0)	25/25 (100.0% CI 86.7–100.0)	73/73 (100.0% 95.0–100.0)	23/28 (82.3% CI 64.4–92.1)	.0012
Serum and/or urine	23/23 (100.0% CI 85.7–100.0)	11/11 (100.0% CI 74.1–100.0)	32/32 (100.0% CI 89.3–100.0)	91/91 (100.0% CI 96.0–100.0)	30/33 (90.9% CI 76.4–96.9)	.0176
BAL	5/5 (100.0% CI 56.6–100.0)	4/4 (100.0% CI 51.0–100.0)	5/6 (83.3% CI 43.7–99.2)	17/18 (94.4% CI 74.2–99.7)	2/5 (40.0% CI 7.1–76.9)	.0209
MVD Ab EIA (>10 EU/mL)						
IgG	9/21 (42.8% CI 24.5–63.5)	3/10 (30.0% CI 10.8–60.3)	24/29 (82.8 CI 65.5–92.4)	56/84 (66.7% CI 56.1–75.8)	24/27 (88.9% CI 71.9–96.2)	.0274
IgM	7/21 (33.3% CI 17.2–54.6)	4/10 (40.0% CI 16.8–68.7)	25/29 (86.2% CI 69.4–94.5)	53/84 (63.1% CI 52.4–72.6)	21/27 (77.8% 59.2–89.4)	.2402
All	11/21 (52.4% CI 32.4–71.7)	5/10 (50.0% 23.7–76.3)	25/29 (86.2% CI 69.4–94.5)	63/84 (75.0% CI 64.8–83.0)	26/27 (96.3% 81.7–99.8)	.0134
MVD Ab EIA (>20 EU/mL)						
IgG	9/21 (42.9% CI 24.5–63.5)	2/10 (20.0% CI 3.6–51.0)	24/29 (82.8% CI 65.5–92.4)	53/84 (63.1% CI 52.4–72.6)	23/27 (85.2% CI 67.5–94.1)	.0343
IgM	2/21 (9.5% CI 1.7–28.9)	2/10 (20.0% CI 3.6–51.0)	20/29 (68.9% CI 50.8–82.7)	34/84 (40.5% CI 30.6–51.2)	18/27 (66.7% CI 47.8–81.4)	.0259
All	10/21 (47.6% CI 28.3–67.6)	3/10 (30.0% CI 10.8–60.3)	24/29 (82.8% CI 65.5–92.4)	57/84 (67.9% CI 57.3–76.9)	24/27 (88.9% CI 71.9–96.2)	.0449
MVD Ag and/or Ab EIA (>10 EU/mL)						
	21/21 (100% CI 84.5–100.0)	10/10 (100% CI 72.3–100.0)	29/29 (100% CI 88.3–100.0)	83/83 (100% CI 95.6–100.0)	26/27 (96.3% CI 81.7–99.8)	.2455
MVD Ag and/or Ab EIA (>20 EU/mL)						
	21/21 (100% CI 84.5–100.0)	10/10 (100% CI 72.3–100.0)	29/29 (100% CI 88.3–100.0)	83/83 (100% CI 95.6–100.0)	26/27 (96.3% CI 81.7–99.8)	.2455
ID (H/M Ab)						
H band	0/7 (0.0% CI 81.7–99.8)	0/5 (0.0% CI .0–43.5)	11/20 (55.0% 34.2–74.2)	14/45 (31.1% CI 19.5–45.7)	7/21 (33.3% CI 17.2–54.6)	1.0000
M band	0/7 (0.0% CI 81.7–99.8)	1/5 (20.0% CI 1.0–62.5)	15/20 (75.0% 53.1–88.8)	22/45 (48.9% CI 35.0–63.0)	15/21 (71.4% CI 50.0–86.2)	.0857
All	0/7 (0.0% CI 81.7–99.8)	1/5 (20.0% CI 1.0–62.5)	16/20 (80.0% 58.4–91.9)	24/45 (53.3% CI 39.1–67.1)	15/21 (71.4% CI 50.0–86.2)	.1637
CF (Yeast/Mycelial)						
Mycelial	1/6 (16.7% CI .9–56.4)	0/2 (0.0% CI .0–82.2)	10/19 (52.6% CI 31.7–72.7)	14/38 (36.8% CI 23.4–52.7)	4/14 (28.6% CI 11.7–54.7)	.7460
Yeast	0/6 (0.0% CI .0–0.0)	0/2 (0.0% CI .0–82.2)	11/19 (57.9% CI 36.3–76.9)	15/38 (39.5% CI 25.6–55.3)	4/13 (30.8% CI 12.7–57.6)	.7432
All	1/6 (16.7% CI .9–56.4)	0/2 (0.0% CI .0–82.2)	12/19 (63.2% CI 41.0–80.9)	18/38 (47.4% CI 32.5–62.7)	5/14 (35.7% CI 16.3–61.2)	.5388
Culture						
Blood	0/20 (0.0% CI .0–16.1)	2/8 (25.0% CI 4.4–59.1)	0/24 (0.0% CI .0–13.8)	2/70 (2.9% CI .5–9.8)	0/19 (0.0% CI .0–16.8)	1.0000
Fungal blood	7/11 (63.6% CI .34–84.8)	4/5 (80.0% CI 37.6–99.0)	4/9 (44.4% CI 18.9–73.3)	15/27 (55.6% CI 37.3–72.4)	1/4 (25.0% CI 1.3–69.9)	.3326
Respiratory (sputum)	0/4 (0.0% CI .0–49.0)	0/3 (0.0% CI .0–56.2)	0/5 (0.0% CI .0–43.5)	0/0 (0.00)	2/9 (22.2% CI 3.9–54.7)	.0952
BAL	6/9 (66.7% CI 35.4–87.9)	3/5 (60.0% CI 23.1–92.9)	4/7 (57.1% CI 25.1–84.2)	19/30 (63.3% 45.5–78.1)	3/10 (30.0% CI 10.8–60.3)	.1401
Pathology						
Histopathology	3/5 (60.0% CI 23.1–92.9)	2/4 (50.0% CI 8.9–91.1)	5/7 (71.4% CI 35.9–94.9)	15/24 (62.5% CI 42.7–78.8)	7/12 (58.3% CI 32.0–80.7)	1.0000
Cytopathology	4/5 (80.0% CI 37.6–99.0)	4/8 (50.0% CI 21.5–78.5)	3/4 (75.0% CI 30.1–98.7)	12/27 (44.4% CI 27.6–62.7)	1/11 (9.1% CI .5–37.7)	.0597

Includes cases only.

^a^χ^2^ or Fisher's exact test used where appropriate.

Immunocompetent individuals were less likely to have a positive *Histoplasma* antigen test 30/33 (90.9% CI 76.4–96.9) when compared to immunocompromised individuals 91/91 (100.0% CI 96.0–100.0) (*P* = .0176). The addition of antibody detection by EIA to antigen testing had a modest improvement in sensitivity at a cutoff of 10 EU/mL when compared to antigen testing alone (Immunocompetent: 26/27, 93.3% vs 30/33, 90.9% *P* = .6199 Immunocompromised: 91/91, 100.0% vs 83/83, 100.0% *P* > .99). Raising the cutoff to 20 EU/mL had no impact on the sensitivity but increased the specificity from 57/79 (72.2% CI 62.3–82.0) to 73/79 (92.4% CI 86.6–98.3 *P* = .0014).

### Pulmonary and Disseminated Disease

Characteristics of diagnostic tests for pulmonary and DH are shown in Table 4.

**Table 4.** Differences in Sensitivity Between Pulmonary and Disseminated Disease

	Pulmonary	Disseminated	*P*-value^a^
			
MVD Ag EIA			
Urine	52/55 (94.6% CI 85.2–98.5)	27/27 (100.0% CI 87.5–100.0)	.5473
Serum	53/55 (94.6% CI 87.7–99.4)	28/28 (100.0% CI 87.9–100.0)	.5475
Serum and/or urine	70/71 (98.6% CI 92.4–99.9)	34/34 (100.0% CI 89.9–100.0)	1.0000
BAL	10/14 (71.4% CI 45.4–88.3)	8/8 (100.0% CI 67.6–100.0)	.2536
MVD Ab EIA (>10 EU/mL)			
IgG	46/61 (75.4% CI 63.3–84.5)	21/31 (67.7% CI 50.1–81.4)	.4645
IgM	43/61 (70.5% CI 58.1–80.4)	16/31 (51.6% CI 34.8–68.0)	.1070
All	49/61 (80.3% CI 68.7–88.4)	23/31 (74.2% CI 56.8–86.3)	.5947
MVD Ab EIA (>20 EU/mL)			
IgG	43/61 (70.5% CI 58.1–80.4)	20/31 (64.5% CI 47.0–78.9)	.6372
IgM	35/61 (57.4% CI 44.9–69.0)	10/31 (32.3% CI 18.6–49.9)	.0283
All	46/61 (75.4% CI 63.3–84.5)	21/31 (67.7% CI 50.1–81.4)	.4645
MVD Ag and/or Ab EIA (>10 EU/mL)			
	60/60 (100.0% CI 94.0–100.0)	31/31 (100.0% CI 89.0–100.0)	N/A
MVD Ag and/or Ab EIA (>20 EU/mL)			
	60/60 (100.0% CI 94.0–100.0)	31/31 (100.0% CI 89.0–100.0)	N/A
ID (H/M Ab)			
H band	14/39 (35.9% CI 22.7–51.6)	2/18 (11.1% CI 2.0–32.8)	.0641
M band	23/39 (59.0% CI 43.4–72.9)	8/18 (44.4% CI 24.6–66.3)	.3059
All	25/39 (64.1% CI 48.4–77.3)	8/18 (44.4% CI 24.6–66.3)	.1623
CF (Yeast/Mycelial)			
Mycelial	11/31 (35.5% CI 21.1–53.1)	2/13 (15.4% CI 2.7–42.2)	.2825
Yeast	11/30 (36.7% CI 21.9–54.5)	4/13 (30.8% CI 12.7–57.6)	1.0000
All	15/31 (48.4% CI 32.0–65.2)	4/13 (30.8% CI 12.7–57.6)	.5069
Culture			
Blood	0/48 (0.0% CI .0–7.4)	2/26 (7.6% CI 1.4–24.1)	.1203
Fungal blood	0/9 (0.0% CI .0–29.9)	12/16 (75.0% CI 50.5–89.8)	.0005
Respiratory (sputum)	1/21 (4.8% CI .2–22.7)	1/6 (16.7% CI .9–56.4)	.4017
BAL	10/24 (41.7% CI 24.5–61.2)	11/13 (84.6% CI 57.8–97.3)	.0165
Pathology			
Histopathology	3/10 (30.0% CI 10.8–60.3)	13/17 (76.5% CI 52.7–94.0)	.0402
Cytopathology	3/18 (16.7% CI 5.8–39.2)	10/15 (66.7% CI 41.7–84.8)	.0052

^a^χ^2^ or Fisher's exact test used where appropriate.

Antigen detection had a high sensitivity in both pulmonary and disseminated disease (70/71, 98.6% CI 92.4–99.9 vs 34/34, 100% CI 89.9–100.0 *P* = 1.00). Antibody detection by EIA was similar in pulmonary and disseminated cases at 10 EU/mL (49/61, 80.3% CI 68.7–88.4 vs 23/31, 74.2% CI 56.8–86.3, *P* = .5947) and 20 EU/mL (46/61, 75.4% CI 63.3–84.5 vs 21/31, 67.7% CI 50.1–81.4 *P* = .4645). Antibody detection was also similar by ID (25/39, 64.1% vs 8/18, 44.4% *P* = .1623) and CF (15/31, 45.2% vs 4/13, 30.8% *P* = .5069). The addition of antigen detection to antibody detection by EIA increased the sensitivity to 60/60 (100% CI 94.0–100.0) for pulmonary disease and 31/31 (100% CI 94.0–100.0) for disseminated disease (*P* = 1.0).

### Acute, Subacute, and CPH

The sensitivity of IgG/IgM by EIA using a cutoff of 10 EU/mL and 20 EU/mL was higher in patients with subacute histoplasmosis (13/13, 100% CI 77.2–100.0; 13/13, 100% CI 77.2–100.0, respectively) and chronic histoplasmosis (3/3, 100% CI 43.9–100.0; 3/3 100% CI 43.9–100.0, respectively) compared to patients with acute histoplasmosis (32/46, 69.5% CI 55.2–80.9; 29/46, 63.0% CI 48.6–75.5, respectively).

The sensitivity of antigen detection by EIA in urine and/or serum was high across all groups (acute: 48/49 98.0% CI 89.3–99.9, subacute: 15/15 100% CI 79.6–100, chronic 5/5 100% CI 89.3–100).

The sensitivity of antibody detection by ID was 13/23 (56.5% CI 36.8–74.4) in APH, 9/11 (81.8% CI 52.3–96.8) in SPH and 2/3 (66.7% CI 11.9–98.3) for CPH. CF was less sensitive with only 9/21 (42.9% CI 24.5–63.5) in APH, 5/7 (71% CI 35.9–94.9) in SPH, and 1/4 (25% CI 1.3–69.9) in CPH.

### Severity of Illness

**Figure 1.**
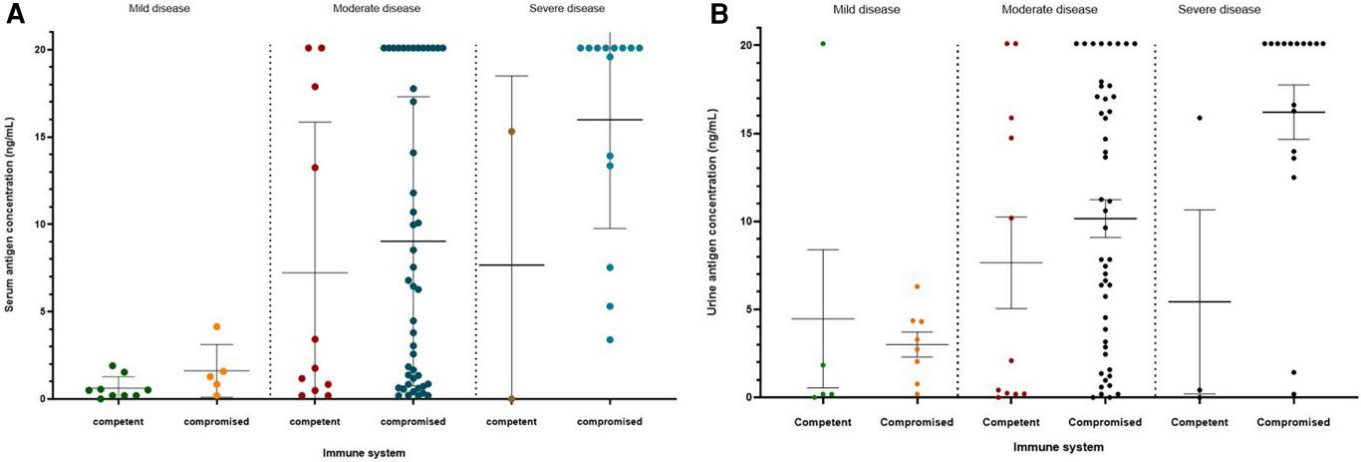
Antigen concentration by disease severity and immune status. *A*, Serum antigen concentrations by EIA. *B*, Urine antigen concentrations by EIA.

When cases were stratified by severity of illness, milder disease was associated with improved sensitivity of serologic testing for ID (11/13 84.6% CI 57.8–97.3 for mild, 18/33 54.5% CI 38.0–70.2 for moderate, and 8/16 50% CI 28.0–72.0 for severe), CF (9/15 60% CI 35.8–80.2 for mild, 9/24 37.5% CI 21.2–57.3 for moderate, and 5/12 41.7% CI 19.3–68.1 for severe), and IgG/IgM EIA (18/19 84.2% CI 82.4–100.0 for mild, 51/63 81.0% CI 69.6–88.8 for moderate, and 13/21 61.9% CI 40.9–79.3 for severe). Combined serum and urine antigen testing remained quite sensitive throughout the spectrum of disease severity (20/21 95.2% CI 77.3–99.8 for mild, 59/59 100% CI 93.9–100 for moderate, and 24/25 96% CI 80.5–99.8 for severe). Fungal isolator blood cultures were more commonly positive in severe cases (6/8 75% CI 40.9–95.6) compared to moderately ill patients (9/22 40.9% 23.6–61.3). The mean concentration of serum antigen in mild, moderate and severe disease was 1.0 +/− 1.1, 8.7 +/− 8.3, and 15.0 +/− 7.0 ng/mL, respectively (*P* < .0001). The mean concentration of urine antigen in mild, moderate and severe disease was 3.6 +/− 5.3, 9.7 +/− 7.6, and 14.6 +/− 7.7 ng/mL, respectively (*P* = .0016) (Table 5 and Figure 1).

**Table 5.** Antigen Concentrations

	Mean Concentration (ng/mL) ± SD			
	Serum	*P*	Urine	*P*
Classification				
Proven	12.9 ± 7.9	.0002	13.2 ± 8.0	.0006
Probable	5.6 ± 7.4		7.2 ± 7.1	
Immune status				
Immunocompromised	9.9 ± 8.3	<.0001	10.8 ± 7.7	.002
SOT	12.8 ± 8.5	.1708	14.0 ± 6.7	.0454
HIV/AIDS	12.1 ± 8.4		14.5 ± 8.4	
TNF-a	7.0 ± 7.1		9.1 ± 7.0	
Immunocompetent	4.2 ± 7.0		5.6 ± 1.9	
Involvement				
Pulmonary	6.4 ± 7.8	.0042	8.6 ± 7.6	.0896
Acute	7.9 ± 8.4	.1264	9.5 ± 7.6	.2925
Subacute	3.7 ± 5.7		6.5 ± 7.4	
Chronic	0.7 ± 0.5		3.3 ± 4.4	
Disseminated	12 ± 8.3		11.5 ± 8.5	
Severity				
Mild	1.0 ± 1.1	<.0001	3.6 ± 5.3	.0016
Moderate	8.7 ± 8.3		9.7 ± 7.6	
Severe/death	15.0 ± 7.0		14.6 ± 7.7	

Concentrations are expressed in ng/mL.

Abbreviation: SD, standard deviation.

## DISCUSSION

In this retrospective study from a tertiary referral center in an endemic region, we comprehensively evaluated the diagnostic performance of multiple laboratory modalities for histoplasmosis, stratified by immune status and clinical syndrome. Our findings affirm the utility of antigen detection, particularly in DH and among immunocompromised hosts, while also demonstrating that IgG/IgM EIA substantially outperforms traditional serologies and offers meaningful diagnostic value in subgroups where antigen performance is more limited.

Urine and serum antigen testing were the most sensitive modalities overall, with urine antigen alone reaching 94.9% sensitivity and combined serum and urine antigen testing achieving 97.6% sensitivity. However, among the 75 patients who underwent both tests, the incremental yield of adding serum to urine antigen detection was modest, improving sensitivity by only 4.5%. This finding indicates that the high combined sensitivity reflects the strong performance of both assays individually, rather than substantial additive benefit. Whether routine dual testing is cost-effective remains uncertain and may depend on clinical context. These results are aligned with the CDC diagnostic algorithm, which recommends urine antigen as the initial diagnostic test for suspected histoplasmosis [7]. In addition, they reinforce the central role of antigen detection in diagnosing histoplasmosis, especially in immunocompromised individuals and patients with DH, where the fungal burden is highest. In patients with DH and APH, combined antigen testing had very high sensitivity (100% and 98.0%, respectively) and remained high in SPH (100%; 95% CI 79.6–100), acknowledging the wide interval due to small numbers. A known limitation of antigen detection is cross-reactivity, most notably with Blastomyces dermatitidis, which should be considered when interpreting positive results in coendemic regions. As has been observed in previous studies, antigen concentrations are significantly higher in patients with DH and in those who are immunocompromised, reflecting the relationship between fungal burden and antigenemia or antigenuria [8, 9]. This observation raises the potential utility of quantitative antigen testing not only for diagnosis but also for risk stratification and monitoring [10].

The sensitivity of antigen testing in SPH was also high (100%; 95% CI 79.6–100), but precision is limited by the small sample size, so additional evaluation is warranted. In this context, IgG/IgM EIA also performed well in SPH (13/13, 100%), consistent with a more mature humoral response. IgG/IgM EIA performed substantially higher than previously reported for traditional serologic methods [4]. These findings suggest that antibody detection by EIA may be useful when antigen results are unavailable, negative/equivocal, or when serologic confirmation is desired, particularly in immunocompetent hosts.

Importantly, our study directly compares the performance of the detection of antibodies by EIA to historical methods such as ID and CF, which have been foundational in histoplasmosis diagnostics for decades [3]. The superior sensitivity and equivalent specificity of EIA underscores the shifting diagnostic landscape and provides empirical support for transitioning away from older serologic assays in favor of newer platforms with higher diagnostic yield. While ID and CF remain embedded in current CDC diagnostic algorithms, accumulating evidence suggests that future revisions may consider incorporating EIA as the preferred serologic modality, pending confirmation from larger multicenter studies.

A major contribution of this study is its syndrome- and host-specific diagnostic stratification, which allows us to propose a nuanced testing approach. For instance, in DH or among immunocompromised hosts, antigen testing remains paramount, with additional testing providing limited incremental yield. In contrast, in immunocompetent patients with SPH, dual testing with antigen and antibody EIA provides the highest sensitivity (100% in our cohort), suggesting a tailored approach based on immune status and clinical presentation.

We also explored antigen levels as a reflection of fungal burden, finding significantly higher concentrations in patients with DH and among immunocompromised hosts. This supports the potential role of quantitative antigen testing for prognostication or therapeutic monitoring [10], though prospective validation is needed.

Culture and histopathology remain valuable diagnostic tools, particularly in confirming histoplasmosis [8]. Fungal isolator blood cultures demonstrated a significantly higher positivity rate (51.6%) compared to standard blood cultures (2.2%), underscoring their utility in disseminated disease. Tissue histopathology was diagnostic in 46.7% of patients, although this may be influenced by selection bias.

Our study has several limitations. As a retrospective analysis, test ordering was clinician-directed and nonstandardized, which may introduce bias. The incorporation of antigen into case definitions introduces incorporation bias. Although incorporation bias is likely to have inflated estimates for antigen, the comparative performance across modalities remains informative. The studied population was not representative of the general population as it is skewed toward patients with immunocompromised conditions and disseminated disease. Furthermore, being a referral center may have shifted the population toward sicker individuals. This may explain the very high sensitivity of *Histoplasma* antigen by EIA across the different groups. Additionally, our findings reflect patients in a high incidence setting with access to advanced diagnostics and may not be generalizable to other regions or practice settings. In addition, the numbers within subgroup analyses were small, and those estimates should be interpreted with caution.

In summary, this study underscores the differential performance of diagnostic tests for histoplasmosis based on clinical presentation and immune status. Antigen detection remains the cornerstone of diagnosis, with either urine or serum providing high sensitivity. The incremental benefit of combining both was modest and may not justify routine dual testing in all patients. The IgG/IgM EIA displayed an improved sensitivity over traditional serologic methods, particularly among immunocompetent individuals with subacute disease, where fungal burden may be lower. These findings support a stratified diagnostic approach tailored to host immune status and clinical syndrome, rather than indiscriminate application of multiple assays. Prospective multicenter studies across diverse epidemiologic settings will be essential to refine cost-effective testing algorithms and validate these observations.
